# Evaluation of Transgenerational Gene Editing Efficiency and Inheritance of Edits Using a Split Cas9/gRNA Crossing System in *Zea mays*

**DOI:** 10.1111/pbi.70391

**Published:** 2025-10-26

**Authors:** Christian Damian Lorenzo, Lennert Impens, Matilde Sanches, Wout Vandeputte, Pieter Wytynck, Stijn Aesaert, Griet Coussens, Dirk Inzé, Hilde Nelissen, Laurens Pauwels

**Affiliations:** ^1^ Department of Plant Biotechnology and Bioinformatics Ghent University Ghent Belgium; ^2^ VIB, Center for Plant Systems Biology Ghent Belgium; ^3^ Department of Biotechnology Ghent University Ghent Belgium

**Keywords:** CRISPR/Cas9, editing efficiency, genetic mosaicism, maize, mutation inheritance, promoter activity, single‐copy events, T‐DNA copy number, transgenerational gene editing

CRISPR/Cas9 genome editing (GE) is used extensively in a wide variety of plant species for research and breeding. However, gene edits observed in somatic cells are not always present in the germline and inherited (Feng et al. 2014). Expression levels of the Cas9 protein are thought to be a main driver. For instance, in *Arabidopsis thaliana* , the use of the CaMV 35S promoter to drive Cas9 expression resulted in poor edit inheritance, which was attributed to weak activity in germline cells and zygotes (Kong et al. 2021). In crops, in somatic tissue sampled from primary transformants (T0s), often more alleles than the ploidy level for a gene are observed. This phenomenon, known as genetic mosaicism, arises from the continued activity of the CRISPR/Cas9 system on different wild‐type alleles that persist through cell divisions and were not edited in the initially transformed cell. Promoters of genes expressed in the germline have been used to drive Cas9 gene expression, enabling edits earlier in plant development, reducing mosaicism and increasing heritability (Rahman et al. 2022). After a GE T0 is pollinated and yields progeny, a single CRISPR/Cas9 T‐DNA locus will show Mendelian segregation. Segregants that lack the CRISPR/Cas9 transgene may have inherited an edited allele. This mutation will be non‐mosaic due to the progression through a single cell (the fertilized egg cell). However, when the CRISPR/Cas9 coding T‐DNA is still present, WT alleles introduced by a cross (or still present from the previous generation) may become edited, creating de novo somatic or germline mutations. This ongoing editing is known as transgenerational gene editing (TGE, Wang et al. 2018). TGE can be exploited to create more genetic variation, edit homoeoalleles in polyploid crops, or edit transformation‐recalcitrant genetic backgrounds (Impens et al. 2022). In our BREEDIT project (Lorenzo et al. 2023), highly multiplex edited maize lines were developed. These lines were generated by supertransforming an Editor line, expressing only Cas9 under the control of the maize *UBIQUITIN* (*pZmUBI*) promoter, with various SCRIPT constructs. Each SCRIPT T‐DNA contains 12 gRNAs driven by alternating rice and wheat U3 promoters. Besides the Mendelian inheritance of edits that occurred in T0, new alleles not observed in T0 leaves were detected in T1. However, only 7% of T1 plants displayed such new edits (Lorenzo et al. 2023), and untangling TGE from Mendelian inheritance was not ideal using this setup.

Here, we used a strategy to specifically evaluate TGE in maize through a genetic cross to combine Cas9 and gRNAs (Figure 1). We first generated new transgenic maize lines in B104 containing only a SCRIPT construct, expressing 12 gRNAs without Cas9 (Figure S1, Table S1). We selected the SCRIPT 2 construct from BREEDIT, which targets nearly all members of the *CYTOKININ OXIDASE* (*CKX*) gene family in maize, as it displayed the highest editing rates among all SCRIPTs (Lorenzo et al. 2023). We selected an event of high quality, SCRIPT 2‐1, with a single T‐DNA copy and no backbone (Table S1) and crossed it with several Editor lines. In addition to Editor 1 (UB‐ED1), the original Editor line used in BREEDIT (Figure S1a), two other independent events from the same transformation experiment (UB‐ED4 and UB‐ED5) were selected, along with one event from another transformation experiment using a construct with a different selection marker (UB‐ED6, Figure S1b). Digital PCR analysis revealed that UB‐ED1 contains four T‐DNA copies, whereas UB‐ED4, UB‐ED5, and UB‐ED6 contain only a single T‐DNA copy and no backbone (Table S1). The SCRIPT 2‐1 × EDs F1 progeny was scored for the presence of both Cas9 and SCRIPT T‐DNAs based on the presence of both selection markers. Because in our split system the parents contain no edits, all observed edits are the result of TGE. The occurrence of edits in seedling leaves is analysed through multiplex amplicon sequencing (MAS) for the 12 *CKX* targets simultaneously (Figure 1a, Figure S2). Following MAS analysis of the F1 generation, we observed TGE in progeny derived from the various Editor lines, calculated as the sum of INDEL haplotype frequencies for each targeted locus (the edit frequency). UB‐ED1 clearly underperformed with an average edit frequency ranging from 0% to 5% for pOsU3‐driven gRNAs and 10%–50% for pTaU3‐driven gRNA compared to 15%–75% (pOsU3) and 80%–100% (pTaU3) for the UB‐ED4, ‐ED5, and ‐ED6 group (Figure 1b). Furthermore, we observed a broader diversity and size range of INDELs (between −68 and +36 bp) for UB‐ED4‐6, while for UB‐ED1 smaller INDELs around −36 and +12 bps were found (Figure S3). Coupled with higher editing rates, UB‐ED4‐6 presented also elevated rates of low‐frequency edits (edits with < 10% frequencies) compared with UB‐ED1, which points towards higher levels of mosaicism (Figure S4a,b). In Arabidopsis, non‐mosaic, heritable mutations were reported using the promoter of the egg cell‐specific gene *EC1*/*DD45* (Kong et al. 2021; Mao et al. 2016), as well as with the promoter of the pollen‐specific gene *SPOROCYTELESS* (Mao et al. 2016). Hence, we generated new constructs with the promoters of the *Brachypodium distachyon* orthologue of rice *BABY BOOM 1 (OsBBM1)*, with expression in sperm cells and early zygote stages (Khanday et al. 2019); and of maize *DNA MEIOTIC RECOMBINASE 1 (ZmDMC1)*, which has meiosis‐associated expression in both male and female lineages (Klimyuk and Jones 1997), to drive Cas9 (Figure S5a,b). The newly generated Editors were also assayed for T‐DNA copy number, and a single copy event was selected for each (BBM1‐ED1 and DMC1‐ED1) and crossed with SCRIPT 2‐1 (Figure 1a, Figures S1a,b and S2, Table S1). F1 plants positive for both BBM1‐ED1 or DMC1‐ED1 and SCRIPT 2‐1 T‐DNAs displayed lower TGE frequencies compared to UB‐ED4‐6 (Figure 1c). However, analysis of the average number of different haplotypes observed per locus showed a marked reduction of low‐frequency edits/mosaicism for BBM1‐ED1 and DMC1‐ED1 versus UB‐ED4‐6 (Figure 1d,e). The use of *pBdDMC1* to drive Cas9 resulted in editing of all targeted *CKX* loci, although the targets of the OsU3‐driven gRNAs displayed reduced editing frequency as was seen in UB‐ED plants. The BdBBM1‐ED1 line showed low efficiencies for TaU3‐driven gRNAs and nearly no TGE for OsU3‐driven gRNAs. Similar results were also observed when testing additional single‐copy events (BBM1‐ED2 and DMC1‐ED2) for both Editors and for another single‐copy event for SCRIPT 2 (SCRIPT 2‐2) (Figure S6). Allele diversity was also markedly different, with DMC1‐ED1 resulting in broader edit outcomes between −53 and +35 bp, while BBM1‐ED1 plants were centred around −33 and +7 bps INDELs (Figure S7). The orientation of genetic crosses (using Editor as male and SCRIPT as female, and vice versa) was not significant in determining TGE frequencies (Figure S8).

**FIGURE 1:**
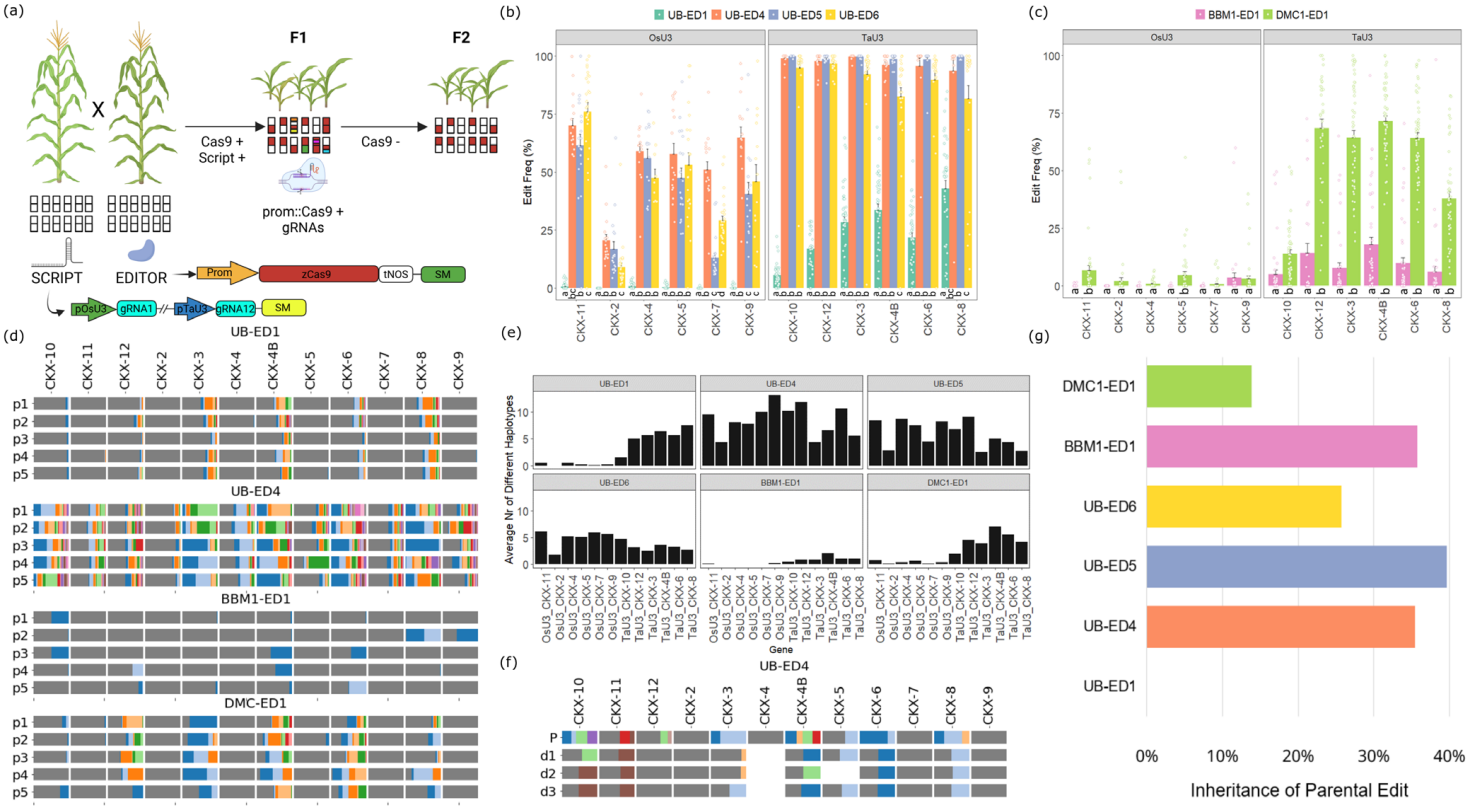
TGE evaluation through the use of a split Cas9/gRNA crossing system. (a) Overview of the strategy. Plants expressing 12 gRNAs (SCRIPT) are crossed with plants only expressing Cas9 (Editors). F1 seedlings with both constructs (Cas9^+^ SCRIPT^+^) are genotyped at the 12 target loci. Coloured bars represent different edited alleles, white bars are reference alleles, and bar lengths are proportional to the allele frequencies. More than two alleles indicate genetic mosaicism. After backcrossing, F2 plants without Cas9 and/or gRNAs are genotyped for inheritance. T‐DNA structures are shown with Prom, the variable promoter driving Cas9; SM, selection marker cassette, zCas9, *Zea mays* optimised Cas9. *tNOS*, *NOPALINE SYNTHASE* terminator, pOsU3: Rice U3 promoter: pTaU3: Wheat U3 promoter. p35S, Cauliflower Mosaic Virus 35S promoter. (b) Edit frequency per locus in F1 plants of Editor lines 1, 4, 5 and 6. Loci separated by gRNA promoter usage. Edit rate of each plant was calculated as the sum of all frequencies of INDEL haplotypes observed for each locus. Bars represent the average (±SE) edit rate on each *locus* for 15–40 plants of each line. Data were analysed using a one‐way ANOVA followed by a Tukey post hoc test. Different letters indicate significant differences (*p*_adj_ < 0.05) among the editor lines (c) Edit frequency per *locus* in F1 plants of lines with tissue‐specific promoters BBM1‐ED and DMC1‐ED. Data represented as in (b), with 40–50 plants of each line. (d) Allele matrices displaying the diversity of INDEL haplotypes in F1 plants of UB‐ED1, UB‐ED4, BBM1‐ED1 and DMC1‐ED1 editors crossed with SCRIPT 2. Each row represent a different plant (p1 to 5) and each column a different locus. Colours represent different alleles within each cell. A total of 5 representative plants per Editor are represented. (e) Edits diversity per locus in F1 plants calculated as the number of distinct INDELs obtained as consequence of TGE in all the plants of each line. Bars represent the average number of distinct haplotypes on each *locus* for 15–50 plants of each line. (f) Allele matrix representing a representative example of inheritance of edits generated through TGE with UB‐ED4. Data representation as in (d). For a F1 parental genotype (P), 3 representative F2 descendants (d) genotypes are shown. White cells indicate missing amplicon sequencing data. (g) Average frequency of edit inheritance in F2 plants of different lines. Degree of inheritance was calculated for each parental edited haplotype as the fraction of plants in the descendance which inherited the parental edit with more than 10% frequency. Only parental edits with more than 10% frequency in F1 were considered. Degree of inheritance were first averaged for each locus (Figure S10), and then all loci were averaged for each line (represented here). Plants with any de novo editing at any gene, and/or positive for the SM, have been removed from the datasets. Bars represent the average edit inheritance across 1–4 progenies per line, with 5–15 F2 plants analysed per progeny.

For all Editors, inheritance of transgenerational edits was subsequently tested in F2 plants after Cas9 and/or SCRIPT out‐segregation through a backcross with B104, avoiding any de novo TGE. The resulting F2 plants were compared to the genotypes of the F1 (Figure 1f,g, Figures S9–S11). Remarkably, ED1 offspring did not inherit any edits from F1, matching low frequencies of somatic transgenerational edits observed in F1 leaves. In contrast, the other UB‐EDs F2 plants inherited a high frequency (15%–80%) of edits (Figure 1g, Figures S9 and S10). BBM1‐ED showed a higher inheritance rate compared to DMC1‐ED, hinting that BBM1‐EDs generate less‐mosaic F1 edits which are more traceable across generations (Figure 1g, Figures S9 and S10).

The distinct editing outcomes of the Editor lines in this panel allow the design of a crossing strategy tailored to the needs of the experimenter (Figure S11). The use of UB‐EDs would allow the generation of a broad mutationally saturated population, adequate to obtain high‐order mutant effects or bypass redundancy. However, our results indicate that high editing frequencies are often accompanied by elevated levels of mosaicism. Instead of doing a de novo transformation, a parallel cross between a SCRIPT line and DMC1‐EDs would then allow an intermediate situation, while a cross with BBM1‐EDs could generate lower‐order mutants, but with high inheritance of edits. In addition, selection of TaU3 promoters to drive gRNAs could enable maximal editing performance through this Editor panel usage.

In conclusion, we show that the event quality (1 copy T‐DNA), the promoters driving Cas9, and the promoter driving gRNAs are all pivotal for high TGE efficiency and ensuring the heritability of edits.

## Author Contributions

C.D.L., L.I., L.P., D.I. and H.N. designed the experiments, and C.D.L., L.I. and W.V. performed the experiments. L.I., G.C. and S.A. transformed maize plants. M.S. and P.W. analysed the data. C.D.L. and L.P. wrote the manuscript with the help of all authors.

## Supporting information


**Appendix S1:** pbi70391‐sup‐0001‐FigureS1‐S12‐TableS1‐S3.docx.

## Data Availability

Data available on request from the authors.
