# Supplementary material for: Evaluation of Transgenerational Gene Editing Efficiency and Inheritance of Edits Using a Split Cas9/gRNA Crossing System in Zea mays
Source: Plant Biotechnol J. 2025 Oct 26;24(3):1500–2. doi: 10.1111/pbi.70391 (PMC12946453; doi:10.1111/pbi.70391)
Supplement: Supplementary file 1 — Appendix S1: pbi70391‐sup‐0001‐FigureS1‐S12‐TableS1‐S3.docx. [file PBI-24-1500-s001.docx]

**Supplementary material**

Lorenzo and Impens et al., 2025

**Methods**

**Cloning of constructs for Editor and SCRIPT lines**

The Cas9 coding sequence containing a *Zea mays*-codon optimized Cas9 was cloned under the control of the *ZmUBIQUITIN-1* (*ZmUBI-1*) promoter and *NOPALINE SYNTHASE* (*NOS)* terminator in pEN-L4-AG-R1 (Houbaert et al., 2018) using GreenGate cloning (Lampropoulos et al., 2013). The transcriptional unit was recombined with pEN-L1-linker-L2 and the pHbm42GW7 destination vector (Karimi et al., 2013). The resulting construct (pXHb-pZmUBIL-Cas9-tNOS) allows maize transformants to be selected with hygromycin. Golden Gate Assembly (based on the GreenGate cloning system) was performed to develop the pMHb-pBdBBM1-zCas9-tnos, pRA-WO-pBdDMC1-zCas9-tNOS and pRA-WO-pZmUBI-zCas9-tNOS (with HRA resistance) expression vectors following the method described by Lampropoulos *et al.*, 2013. The latter contain a morphogenic gene cassette (MGC) which allows Cre-mediated auto-excision flanked by loxP sites following the same setup detailed in Aesaert and col (Aesaert et al 2022). To identify the promoters of *BdBBM1* and *BdDMC1*, we chose the *Brachypodium distachyon* genes closest related to rice *BBM1* and maize *DMC1* (Fig S12) using PLAZA (Van Bel et al., 2022) and cloned their promoter sequence to drive the expression of *Cas9.* We selected the promoter sequence based on data from the *Brachypodium* Plant DNase I hypersensitive Sites Database (PlantDHS, <http://plantdhs.org/Jbrowse/Brachypodium>) and checked their expression patterns in Brachypodium gene expression EFP browser (https://bar.utoronto.ca/, Fig S5 a, b). For the promoter sequences, 916 bp before the start codon of *BdBBM1* and 927 bp before the start codon of *BdDMC1* were cloned using Golden Gate cloning to drive the expression of *Cas9*. For SCRIPT 2 construct, the same vector used in Lorenzo et al was used without changes (Lorenzo et al 2023). In this vector design, the initiating G for the sgRNA when using a U3 promoter is part of the Golden Gate overhang. Hence, all sgRNAs will start with an ‘extra’ G, resulting in a GN20 start of the sgRNA with N20 the sequence listed in Table S2. Previous studies have reported that in plants such GN20 design does not impact efficiency for WT Cas9 (Zhang et al., 2017). The maps for all vectors can be downloaded from PSB vector vault (https://vectorvault.vib.be/).

**Maize transformation and selection of single-copy events**

Maize transformation for pXHb-pZmUBIL-Cas9-tNOS and pMHb-pBdBBM1-zCas9-tnos (hygromycin resistance) was performed as described in Vandeputte et al., 2024. Transgenics lines with pRA-WO-pBdDMC1-zCas9-tNOS and pRA-WO-pZmUBI-zCas9-tNOS which have Imazapyr resistance and SCRIPT 2 B104 with Bialaphos resistance were all generated following the protocol of Aesaert et al., 2022. For all events obtained, dPCR was performed and at least two different 1-TDNA copy events without backbone and morphogenic gene cassette, were used for all assays (Table S2).

**Digital PCR**

For copy number analysis, digital PCR (dPCR) was performed using the QIAcuity Digital PCR System (Qiagen). All steps described were performed in a pre-PCR environment to avoid contamination with highly concentrated PCR products or plasmids. Multiplex PCR with IDT probes uses 6-FAM, HEX and Cy5 fluorophores for gene of interest, reference gene, and a T-DNA vector backbone gene respectively. For confirming auto-excision of the MGC, primers and probe (6-FAM) against MoCRE were used in a separate dPCR. Genomic DNA (gDNA) from plants of interest was extracted using the Wizard® Genomic DNA Purification kit (Promega) and measured using Qubit dsDNA HS Assay Kit (Qubit). Maize gDNA was diluted to 5 ng/μL for every sample. A PCR reaction was carried out using a master mix containing per sample, 3.5 μL PCR mix (Qiagen), 1.4 μL of each primer/probe-mix (FAM, HEX, Cy5; each individual primer/probe-mix contains 10 μM Fw primer, 10 μM Rv primer, 5 μM probe, Table S3), 0.5 μL (5U) CviQI restriction enzyme, 4.8 μL H_2_O, 1 μL gDNA (5 ng/μL) for a total volume of 14 μL. Samples and master mix were brought into 8-strip PCR tubes and mixture is incubated for 10-15 min at 24°C. Then, Editors and SCRIPT samples were transferred to a 24-well 26K and 8.5K plate dPCR Nanoplates respectively (Qiagen), the plates were tightly sealed and dPCR reaction was run in the QIAcuity dPCR machine. QIAcuity Software Suite is used to analyze the data and determine copy number variation within the samples.

**Detached leaf assay to determine presence of the selection markers.**

A detailed scheme describing the TGE strategy is presented in Figure S2. Seeds resulting from crossing SCRIPT2 lines with the different Editor lines (F1) were grown in jiffy Peat Pellets (<https://jiffygroup.com/>) and germinated seedlings were grown until V2. For phosphinothricin and hygromycin resistance determination, 1cm pieces of leaf 1 or leaf 2 were placed in 24 well plates containing MS media supplemented with 0.5 mg 6-BAP and 100 mg filter-sterilized hygromicin (for presence of the hygromycin phosphotransferase (HPT) gene) or 6 mg filter-sterilized phosphinothricin (PPT, for BAR presence). Plates were laid to rest for 4 days and resistant plants were scored based on tissue survival. For UB-ED4 (*HRA* selection marker) , PCRs were performed using Cas9 primers detailed in Table S3.

**Genomic DNA isolation, Sanger sequencing, and multiplex amplicon sequencing**

A piece of 1-2 cm of leaf material was placed in 8-strip, 2-mL capacity tubes (National Scientific Supply Co) together with two 3-mm stainless steel ball bearings, snap frozen in liquid nitrogen, and ground using a Mixer Mill MM400 (Retsch®). 0.5 mL DNA extraction buffer (2.5 mL 1 M Tris-HCl pH 8, 3 mL 5 M NaCl, 5 g saccharose, to 50 mL with Milli-Q water) was added, and samples were shaken and incubated at 65°C for 20 min. Tubes were centrifuged (2 min at 1800 x g) and 50 µL of supernatant was mixed with 70 µL magnetic beads (HighPrep™ PCR Clean-up System, Magbio) and put on a magnet. The supernatant was taken off, beads were washed twice with 80% ethanol and dried for further processing.

**Amplicon sequencing for edit detection**

Amplicon sequencing was performed as previously described (Lorenzo *et al*. 2023). HiPlex library preparation was performed by Floodlight Genomics facility (Knoxville, TN, USA).

**Editing rate determination in F1 and F2**

Edit rate of each plant was calculated as the sum of frequencies of all INDEL haplotypes observed for each locus following the methodology used in Lorenzo et al 2023. In F2, all haplotypes were always considered, even if with low frequency (<10%) in which case is highly likely to be (though not exclusively) somatic editing. For calculations of inheritance, haplotypes with frequency lower than 10% were excluded from both F1 (parental) and F2 (progeny). Degree of inheritance was calculated for each parental edited haplotype as the fraction of plants in the progeny which inherited a parental edit with more than 10% frequency.

**References**

**Aesaert, S., Impens, L., Coussens, G., Van Lerberge, E., Vanderhaeghen, R., Desmet, L., Vanhevel, Y., Bossuyt, S., Wambua, A.N., Van Lijsebettens, M., Inzé, D., De Keyser, E., Jacobs, T.B., Karimi, M., and Pauwels, L.** (2022). Optimized Transformation and Gene Editing of the B104 Public Maize Inbred by Improved Tissue Culture and Use of Morphogenic Regulators. Front Plant Sci **13,** 883847.

**Houbaert, A., Zhang, C., Tiwari, M., Wang, K., de Marcos Serrano, A., Savatin, D.V., Urs, M.J., Zhiponova, M.K., Gudesblat, G.E., Vanhoutte, I., Eeckhout, D., Boeren, S., Karimi, M., Betti, C., Jacobs, T., Fenoll, C., Mena, M., de Vries, S., De Jaeger, G., and Russinova, E.** (2018). POLAR-guided signalling complex assembly and localization drive asymmetric cell division. Nature **563,** 574-578.

**Karimi, M., Inzé, D., Van Lijsebettens, M., and Hilson, P.** (2013). Gateway vectors for transformation of cereals. Trends in Plant Science **18,** 1-4.

**Lampropoulos, A., Sutikovic, Z., Wenzl, C., Maegele, I., Lohmann, J.U., and Forner, J.** (2013). GreenGate - A novel, versatile, and efficient cloning system for plant transgenesis. Plos One **8,** e83043.

**Lorenzo, C. D., Debray, K., Herwegh, D., Develtere, W., Impens, L., Schaumont, D., Vandeputte, W., Aesaert, S., Coussens, G., De Boe, Y., et al.** (2023). BREEDIT: a multiplex genome editing strategy to improve complex quantitative traits in maize. *The Plant Cell* **35**:218–238.

**Van Bel, M., Silvestri, F., Weitz, E.M., Kreft, L., Botzki, A., Coppens, F., and Vandepoele, K.** (2022). PLAZA 5.0: extending the scope and power of comparative and functional genomics in plants. Nucleic Acids Research **50,** D1468-D1474.

**Vandeputte, W., Coussens, G., Aesaert, S., Haeghebaert, J., Impens, L., Karimi, M., Debernardi, J.M., and Pauwels, L.** (2024). Use of GRF‐GIF chimeras and a ternary vector system to improve maize (Zea mays L.) transformation frequency. The Plant Journal **119,** 2116-2132.

**Zhang, D., Zhang, H., Li, T., Chen, K., Qiu, J.-L., and Gao, C.** (2017) Perfectly matched 20-nucleotide guide RNA sequences enable robust genome editing using high-fidelity SpCas9 nucleases. Genome Biol, 18, 191

**Supplementary Figures and tables**

a

**
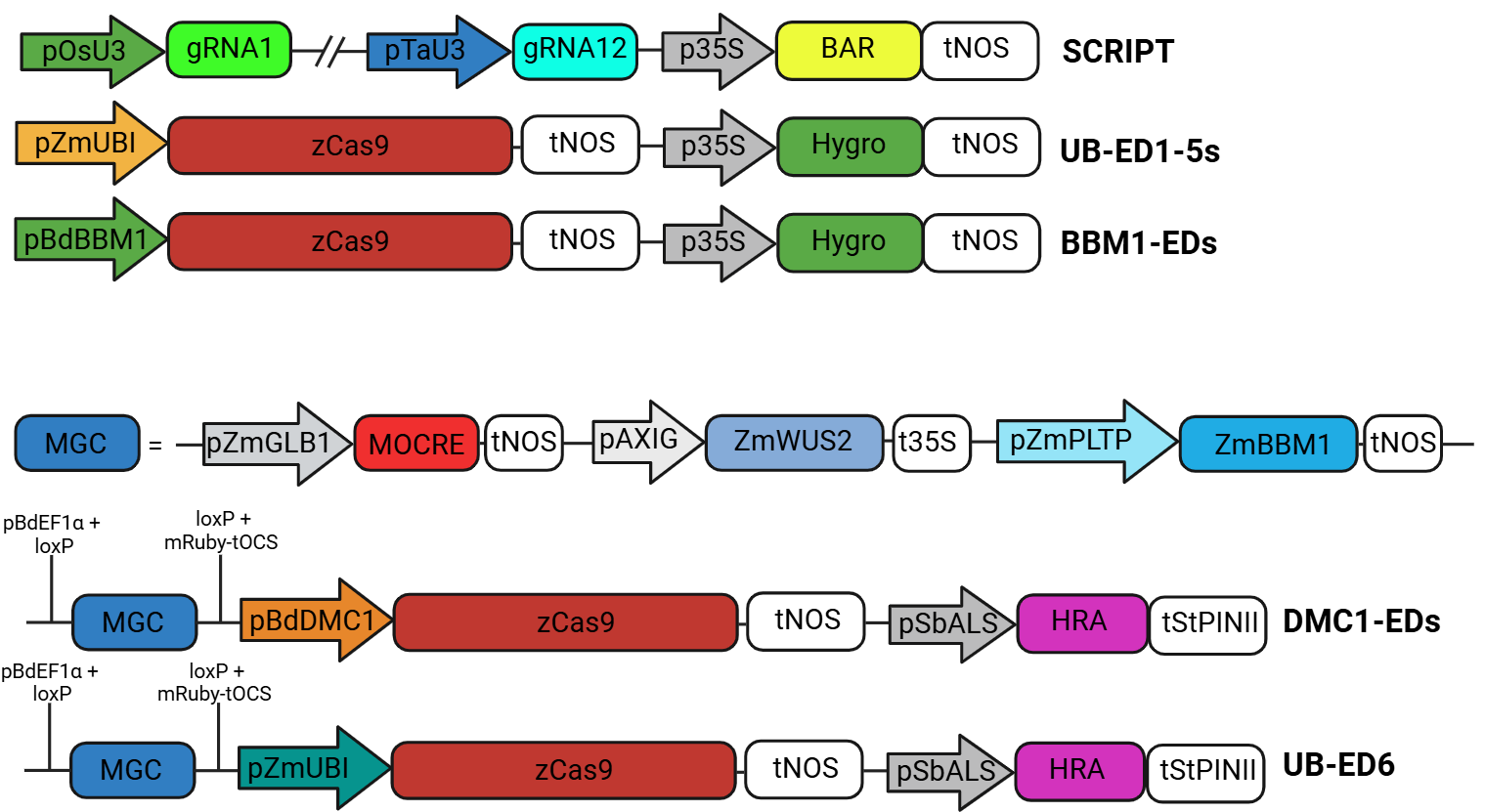
**

b

**Fig S1:** Simplified schematic representation of the T-DNA structure of SCRIPT and all Editor lines used in this study. (a): Structure of SCRIPT, UB ED1 to 5 and BBM1-ED. (b): Structure of DMC1-ED and UB-ED6 transformed in maize using a morphogenic gene cassette (MGC*pZmGLB1*: promoter sequence of ABA-inducible maize *GLOBULIN-1* gene, MoCRE: monocot optimized CRE recombinase, *pAXIG*: *ZmIAA25* auxin-inducible promoter, *ZmWUS*: maize *WUSCHEL2* gene, *pZmPLTP*: maize *PHOSPHOLIPID TRANSFER PROTEIN* promoter, *ZmBBM1*: maize *BBM1* gene, *tStPINII*: *Solanum tuberosum* *PinII* terminator. p35S: 35s promoter, *pSbALS*: *Sorghum bicolor* *ACETOLACTATE SYNTHASE* promoter; HRA: *HIGHLY-RESISTANT ACETOLACTATE SYNTHASE. tNOS: NOPALINE SYNTHASE* terminator*. Cas9:* maize codon optimized Cas9. pOsU3, rice U3 RNA polymerase III promoter; pTaU3, wheat U3 RNA polymerase III promoter; BAR, *BIALAPHOS RESISTANCE*. Hygro: *HYGROMYCIN PHOSPHOTRANSFERASE*. gRNA: guide + scaffold sequence. pZmUBI: *Zea mays* Ubiquitin promoter. pBdBBM1: *Brachypodium distachyon* *BBM1* promoter. pBdDMC1: *Brachypodium distachyon* *DMC1* promoter. LoxP: Lox P recombination sites, LoxP site,: pBdEF1α: *Brachypodium distachyon* *ELONGATION FACTOR 1 ALPHA* promoter. tOCS: *OCTOPINE SYNTHASE* terminator. Maps including detailed T-DNA structures for all vectors can be downloaded from Vectorvault (https://vectorvault.vib.be/).


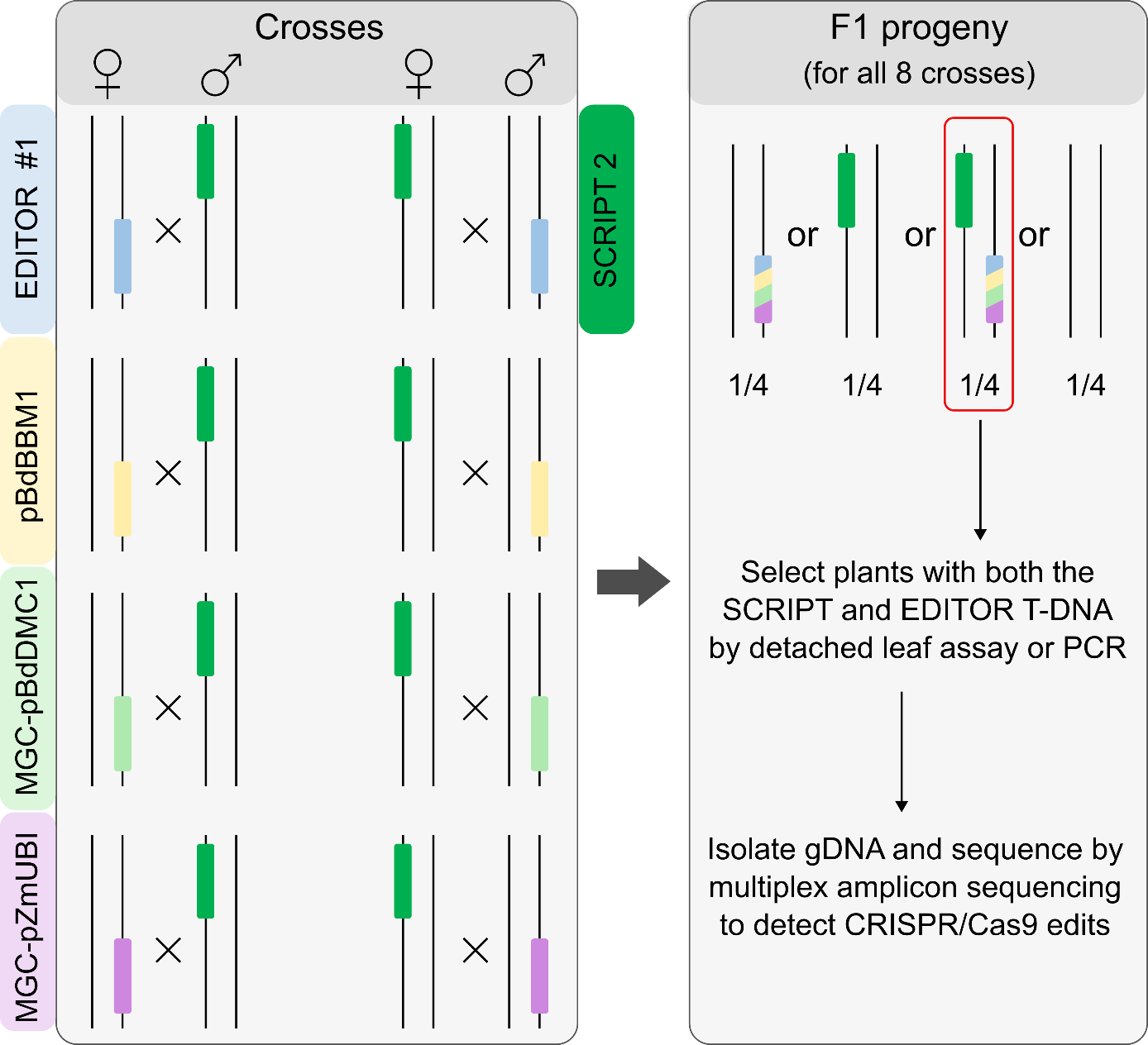


UB-ED6

UB-ED1-4

DMC1-ED

BBM1-ED

**Figure S2.** Visual detailed summary of the crossing and selection process of Editors and SCRIPT T-DNA lines. Heterozygous single copy lines of each Editor (except UB-ED1 with 4 T-DNA copies in one locus) were crossed with heterozygous SCRIPT 2 single copy lines in both directions. F1 progeny carrying both transgenes was selected for gDNA isolation and multiplex amplicon sequencing analysis.

**
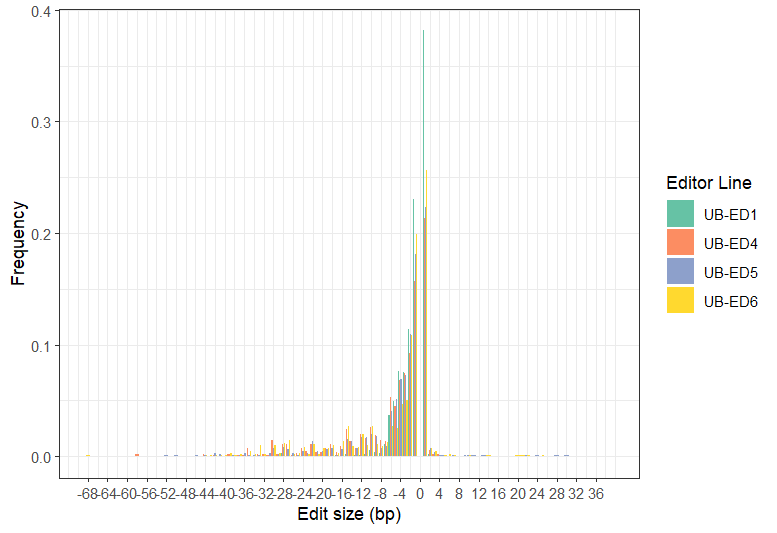
**

**Fig S3:** Allele diversity observed in F1 progenies from UB-ED1 to 6 crossed with SCRIPT 2-1. Different colors represent different Editor lines.

a

a


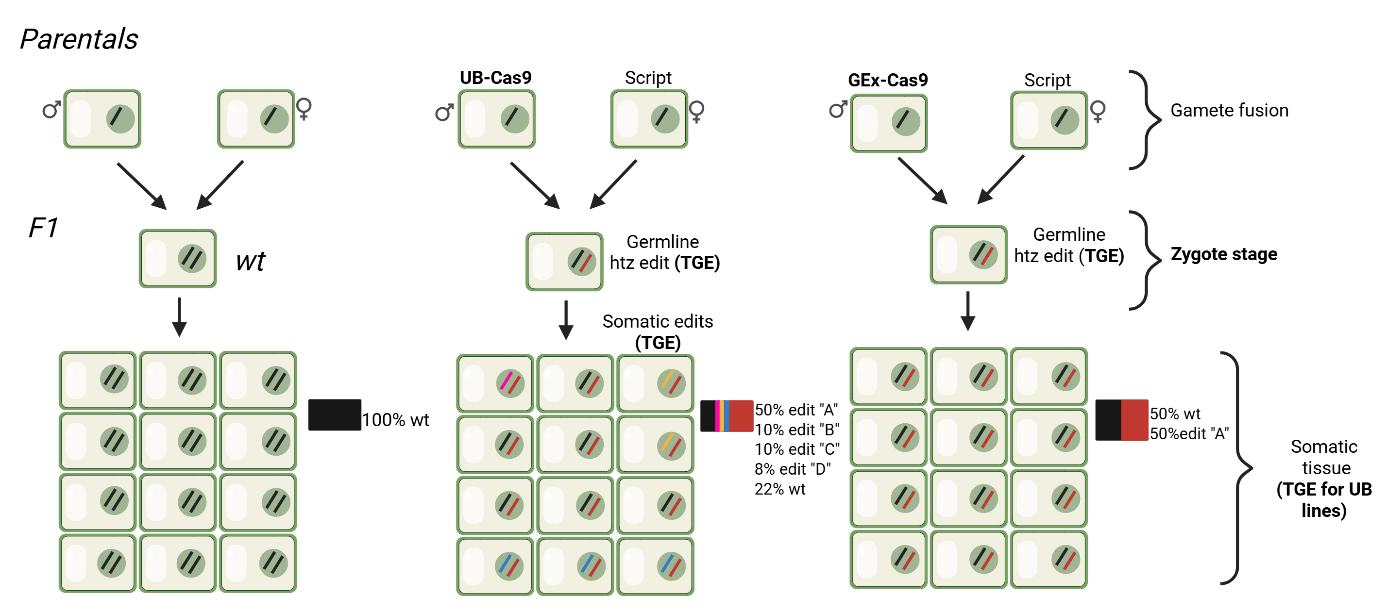


**
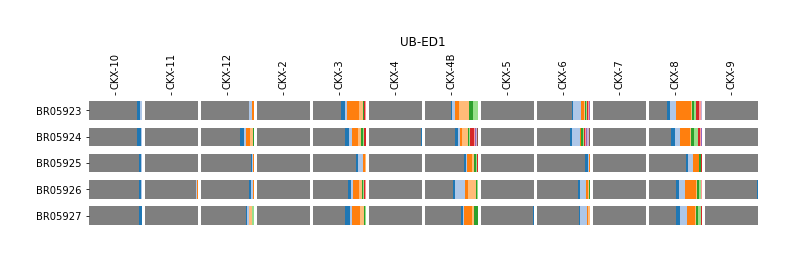
**

b

**
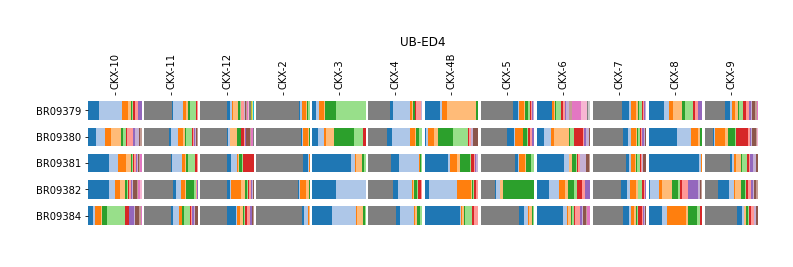
**

**
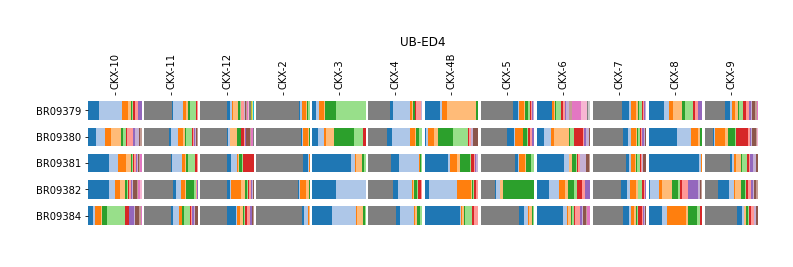
**

**
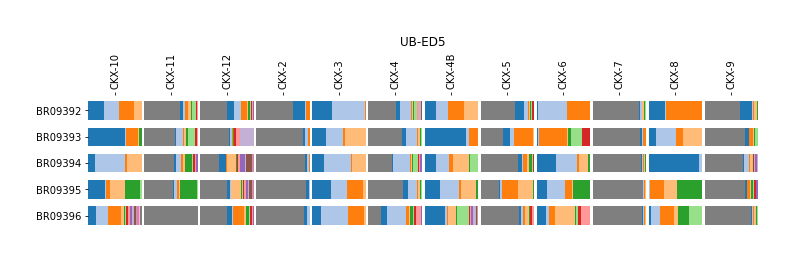
**

**
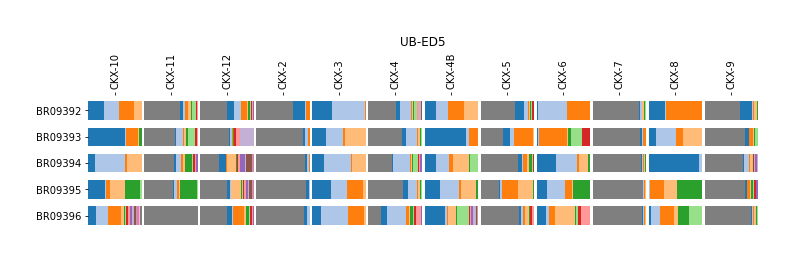
**

**
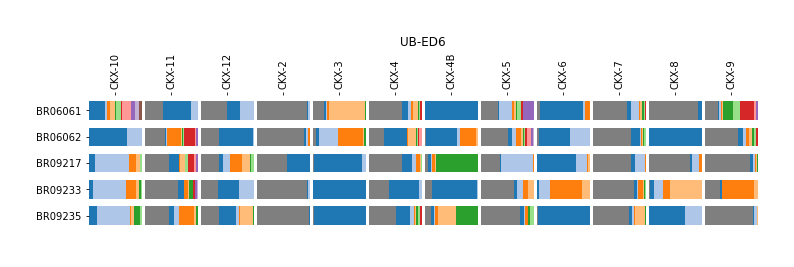
**

**
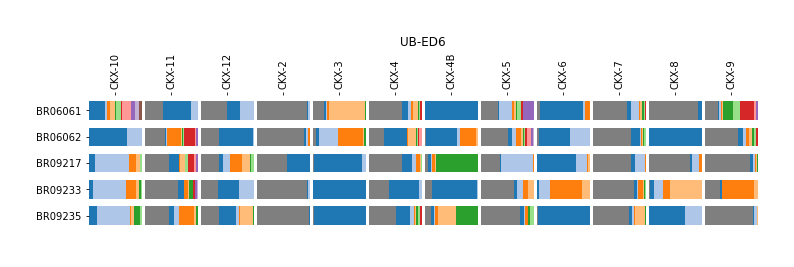
**

**Fig S4:** Allele contributions observed in genotypes of TGE F1 Cas9+SCRIPT+ plants. (a) Schematic representation of edit occurrence through TGE and allele matrix buildup. The situation on the left shows wild-type; the middle shows a UB-ED line with TGE at both the zygote and later stages; and the right shows promoters with germline expression (GEx) (b) Allele matrices exemplifying the diversity of INDEL haplotypes in F1 plants of UB-ED1, UB-ED4, UB-ED5 and UB-ED6 TGE induced progenies. Each row represents a different plant and each column a different locus. Colors represent different alleles within each plant.

**Fig. S5:** Gene expression for *BdBBM1* and *BdDMC1*. (a) *BdBBM1* and (b) *BdDMC1* expression in reproductive tissue. Figure taken from *Brachypodium* eFP browser (https://bar.utoronto.ca/).


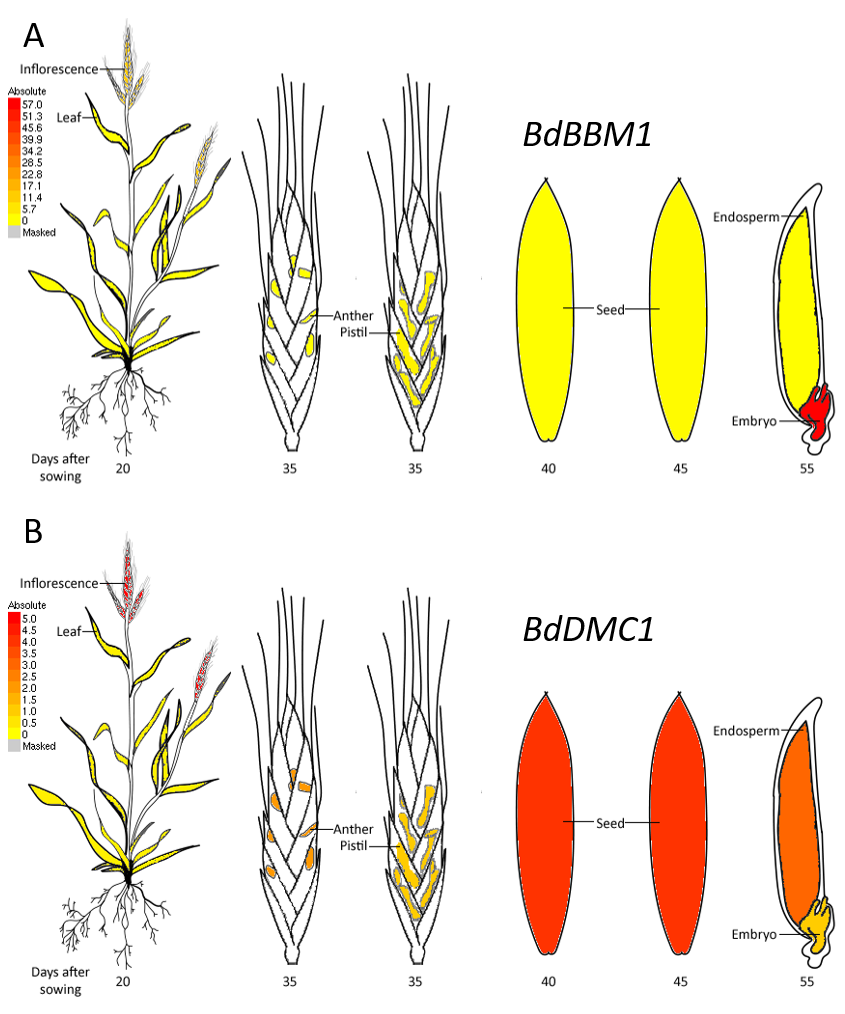


a

b


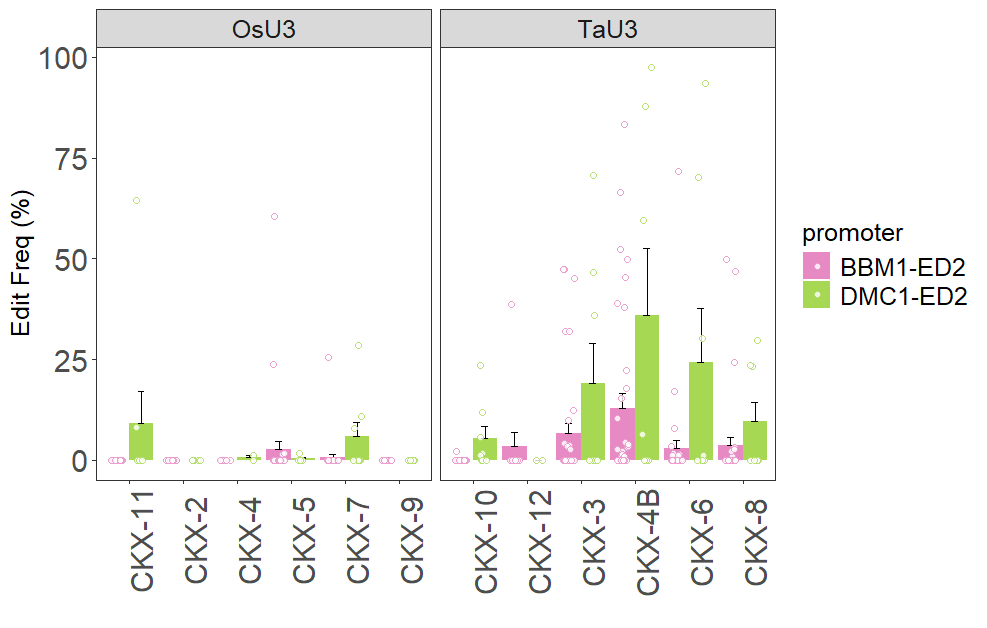


a

a

a

a

a

a

a

a

a

a

a

b

a

a

a

a

a

a

a

b

a

a

a

a

**Fig S6:** Edit freq. per locus in F1 plants of lines with additional events of Editors BBM1-ED2 and DMC1-ED2 crossed with and independent event of SCRIPT 2 (SCRIPT 2-2). Loci are separated by U3 promoter driving gRNA expression. Edit rate of each plant was calculated as the sum of all frequencies of INDEL haplotypes observed for each locus. Bars represent the average (±std.error) edit rate on each locus for 3-30 F1 plants of each Editor line. Data were analyzed using a one-way ANOVA followed by a Tukey post hoc test. Different letters indicate significant differences (padj<0.05) among the editor lines

**
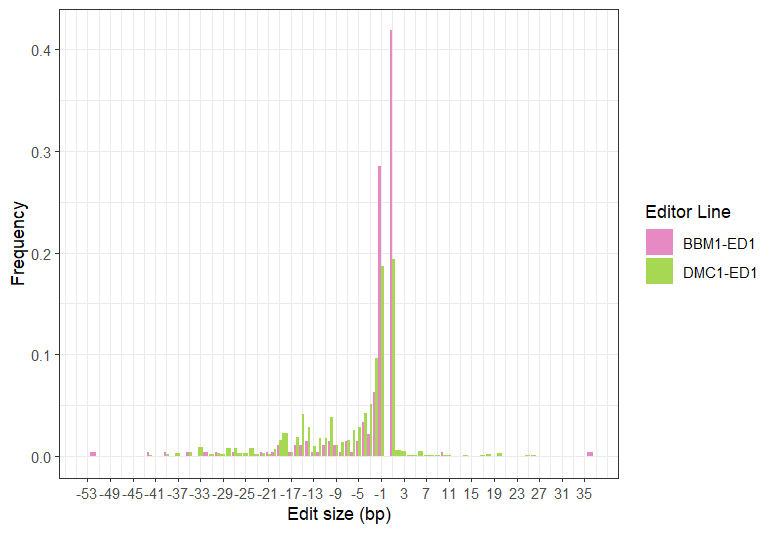
**

**Fig S7:** Allele diversity observed in F1 progenies from BBM1-EDs and DMC1-EDs crossed with SCRIPT 2. Different colors represent different Editors.


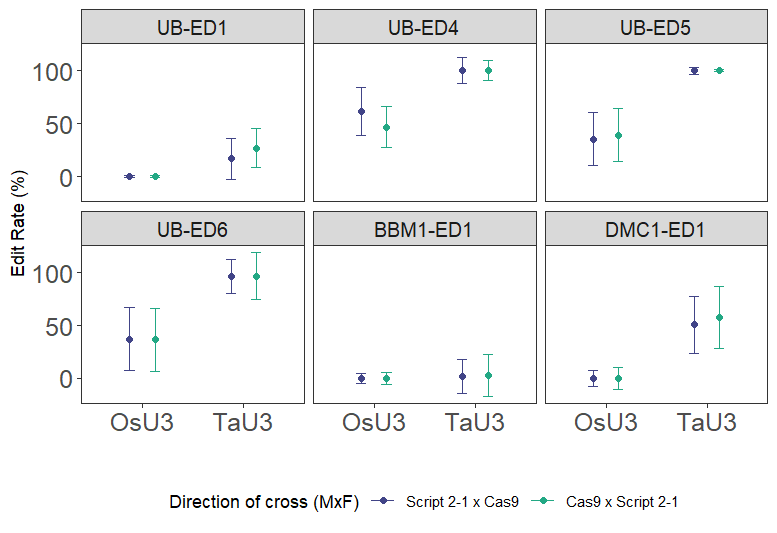


**Fig S8** **:** Mean (±std.deviation) Editing rates observed by Editor line and discriminated by U3 Promoter usage per cross orientation. The middle point represents mean values and error bars stand for standard deviation.


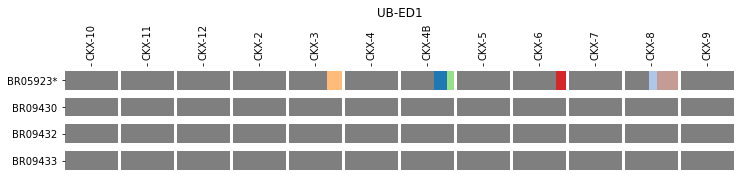

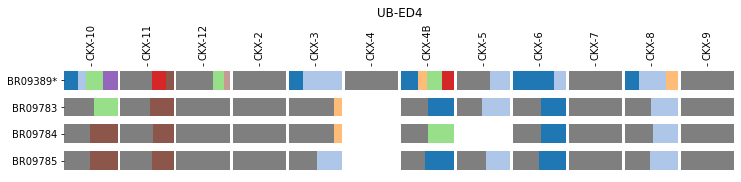

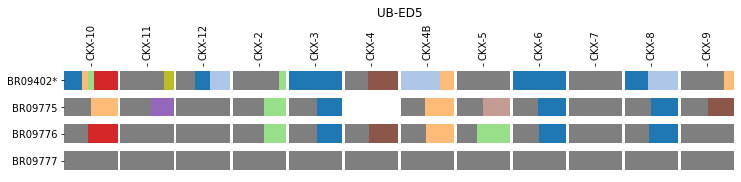

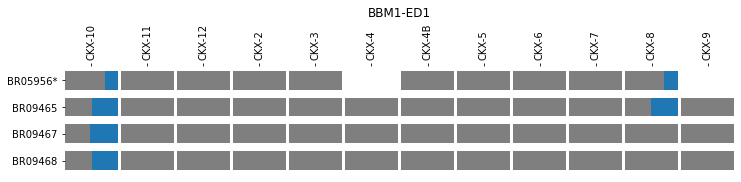

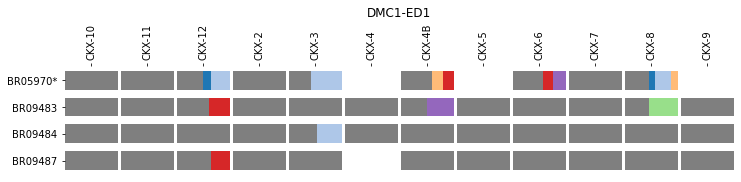


**Fig S9:** Example of allele matrices representing inheritance of edits in F2 generated through TGE with UB-ED1, UB-ED4, UB-ED5, BBM1-ED and DMC1-ED. Each row represents a different plant and each column a different locus. Colors represent different alleles inside each Editor matrix. For each matrix * Indicate parental line genotype and 3 descendants per parent are shown. White cells indicate missing amplicon sequencing data.


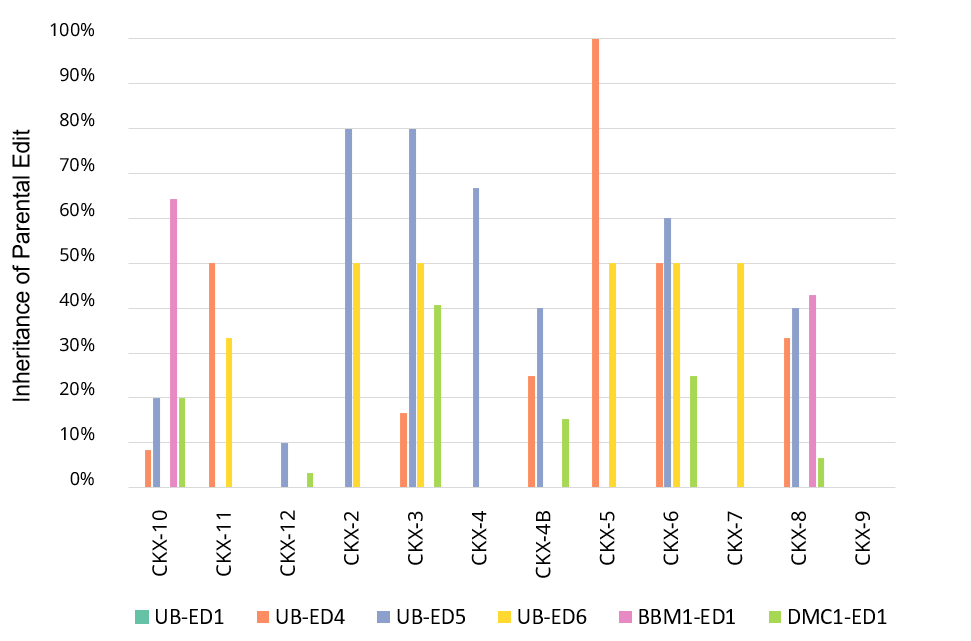


**Fig S10:** Average rate of edits inheritance in F2 plants of different lines. Rate of inheritance was calculated for each parental edited haplotype as the fraction of plants in the descendance which inherited the parental edit with more than 10% frequency. Only parental edits with more than 10% frequency in F1 were considered; rates of inheritance averaged for each locus. Plants with any *de novo* editing at any gene, and/or positive for the presence of the Basta and/or hygromycin selection marker, have been removed from the analysis. Bars represent the average edit inheritance of 2-4 F2 progenies analyzed per line, each composed of 5-15 plants.


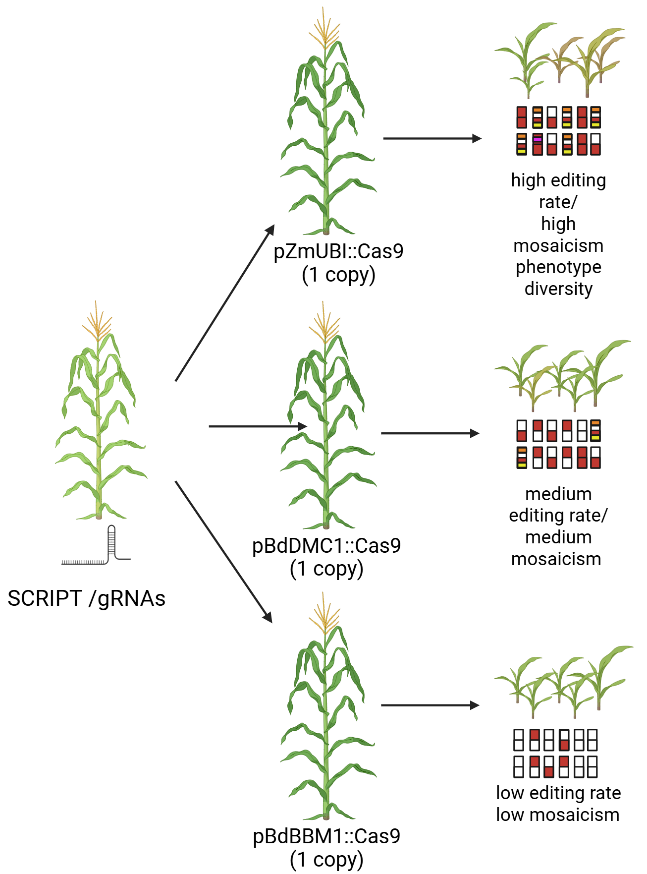


**Fig. S11:** Proposed model exploiting Editor panel diversity. A line carrying gRNAs can be used as pollen donor to pollinate different lines for different editing output. Crosses with pZmUBI::Cas9 (e.g. UB-ED4) will yield high edited saturated genotypes with high mosaicism rates. *pBdDMC1*:Cas9 (DMC1-ED) will render an intermediate approach with reduced mosaicism at the expense of reduced saturation. Finally, *pBdBBM1*::Cas9 (BBM1-ED) will result in low order editing useful for low order mutant identification and validation.


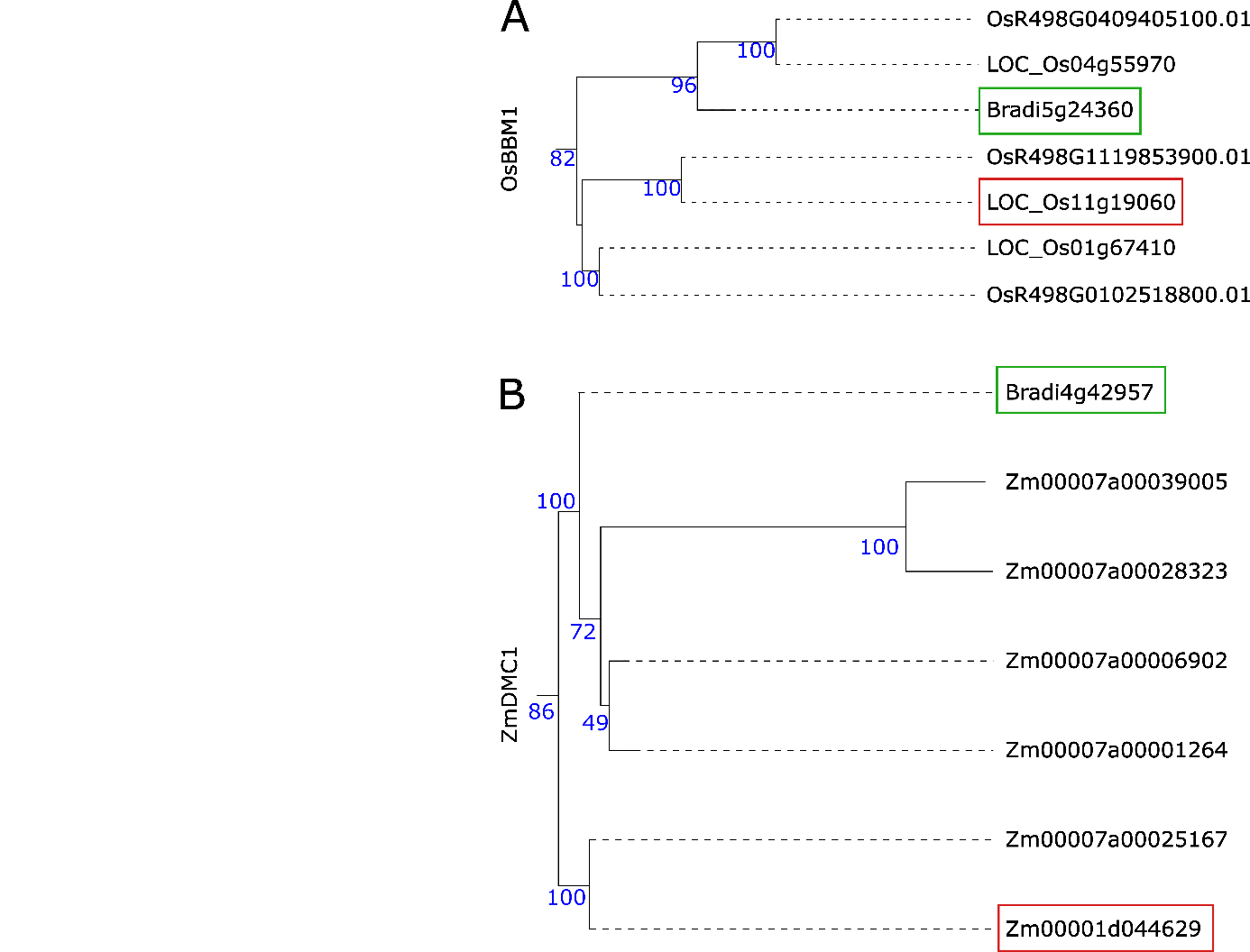


a

b

**Figure S12:** Phylogenetic tree of (A) *OsBBM1* and (B) *ZmDMC1*. Red boxes indicate the seed gene for which the tree was constructed, and green boxes indicate the closest related *Brachypodium* gene. The phylogenetic tree was constructed using PLAZA (Van Bel *et al.*, 2022).

**Table S1:** Copy number of Cas9 and backbone (Aada spectinomycin resistance gene) transgenes for all Editor line events generated measured by dPCR. * Indicate events further characterized in this study

| **Name** | **ID code** | **Construct** | **Resistance marker** | **Zygosity** | **T-DNA Copy N° (per N)** | **Backbone Copy N° (per N)** |
| --- | --- | --- | --- | --- | --- | --- |
|  |  |  |  |  |  |  |
| UB-ED1 | 2019-532-26 | pZmUBI::CAS9 (Editor 1)* | HPT | homozygous | 4 | 4 |
| UB-ED2 | 2019-536-50 | pZmUBI::CAS9 (Editor 2) | HPT | homozygous | 3 | 0 |
| UB-ED3 | 2019-536-62 | pZmUBI::CAS9 (Editor 3) | HPT | homozygous | 3 | 0 |
| UB-ED4 | 2019-536-56 | pZmUBI::CAS9 (Editor 4)* | HPT | homozygous | 1 | 0 |
| UB-ED5 | 2019-536-57 | pZmUBI::CAS9 (Editor 5)* | HPT | homozygous | 1 | 0 |
| UB-ED6 | 711-374-G | pZmUBI::CAS9 (Editor 6 )* | HRA | heterozygous | 1 | 0 |
| S2- 1 | 830-371-A | SCRIPT 2 B104 (A)* | Basta | heterozygous | 1 | 0 |
| S2- 2 | 830-371-Y | SCRIPT 2 B104 (Y)* | Basta | heterozygous | 1 | 0 |
| S2- 3 | 830-371-D | SCRIPT 2 B104 (D) | Basta | heterozygous | 4 | 0 |
| S2- 4 | 830-371-F | SCRIPT 2 B104 (F) | Basta | heterozygous | 2 | 1 |
| S2- 5 | 830-371-O | SCRIPT 2 B104 (O) | Basta | heterozygous | 1 | 0 |
| S2- 6 | 830-371-B | SCRIPT 2 B104 (B) | Basta | heterozygous | 2 | 0 |
| S2- 7 | 830-371-AH | SCRIPT 2 B104 (AH) | Basta | heterozygous | 4 | 0 |
| S2- 8 | 830-371-AJ | SCRIPT 2 B104 (AJ) | Basta | heterozygous | 5 | 3 |
| DMC1-ED1 | 703-388-Aa | pBdDMC1::CAS9 (Aa)* | HRA | heterozygous | 1 | 0 |
| DMC1-ED2 | 703-388-Ab | pBdDMC1::CAS9 (Ab)* | HRA | heterozygous | 1 | 0 |
| BBM1-ED1 | 748-428-A | pBdBBM1::CAS9 (A) | HPT | heterozygous | 2 | 0 |
| BBM1-ED2 | 748-428-Ba | pBdBBM1::CAS9 (Ba)* | HPT | heterozygous | 1 | 0 |
| BBM1-ED3 | 748-428-Ca | pBdBBM1::CAS9 (Ca)* | HPT | heterozygous | 1 | 0 |
| BBM1-ED4 | 748-428-D | pBdBBM1::CAS9 (D) | HPT | heterozygous | 2 | 0 |
| BBM1-ED5 | 748-428-Ea | pBdBBM1::CAS9 (Ea) | HPT | heterozygous | 9 | 0 |
| BBM1-ED6 | 748-428-F | pBdBBM1::CAS9 (F) | HPT | heterozygous | 11 | 2 |
| BBM1-ED7 | 748-428-H | pBdBBM1::CAS9 (H) | HPT | heterozygous | 9 | 3 |
| BBM1-ED8 | 748-428-Jb | pBdBBM1::CAS9 (Jb) | HPT | heterozygous | 1 | 0 |

**Table S2: Target genes, gRNAs and type of U3 promoter allocated in SCRIPT 2. PAM sequences are indicated in red. The initiating G for the U3 promoter is part of the Golden Gate overhang in the vector.**

| **Gene name** | **Identifier (B73V3)** | **gRNA sequence 5’-3’ (+PAM sequence)** | **U3 promoter** |
| --- | --- | --- | --- |
| CKX-10 | GRMZM2G348452 | AGGCCCCGCACGTGCTTGAG CGG | TaU3 |
| CKX-11 | GRMZM2G122340 | TCCTCTGCAGGTTGTGGACC TGG | OsU3 |
| CKX-12 | GRMZM2G008792 | TTGGGCGGAGCAACTTGCAG CGG | TaU3 |
| CKX-2 | GRMZM2G050997 | ATAGCACTCTTCCATCTGAC TGG | OsU3 |
| CKX-3 | GRMZM2G167220 | TGAGACGCTCAAGCACGGTC TGG | TaU3 |
| CKX-4a | GRMZM5G817173 | CTTGAGCGAGGTGAGCTTCA CGG | OsU3 |
| CKX-4 | GRMZM2G024476 | TGTTGTGGCTGTTACCCTTT GGG | TaU3 |
| CKX-5 | GRMZM2G325612 | TTCCTGGACCGCGTGAGCGC CGG | OsU3 |
| CKX-6 | GRMZM2G404443 | CTTCCTGAACCGGGTCAGGA TGG | TaU3 |
| CKX-7 | GRMZM2G114427 | TGTTCATGGCAGCTCTAGGC GGG | OsU3 |
| CKX-8 | GRMZM2G162048 | GAACTCGGACCTGTTCATGG CGG | TaU3 |
| CKX-9 | GRMZM2G303707 | CACCCAATGCTGCGTAGAAC AGG | OsU3 |

**Table S3: Primers used in this study**

| **Name** | **Sequence (5'-3')** | **Description** | **Direction** |
| --- | --- | --- | --- |
| **PCR / cloning** |  |  |  |
| CROPGEN604 | TGTggtctcaacctAGACGCTGTACAACCATCATGT | BdDMC1 | Fw |
| CROPGEN605 | TGTggtctcatgttCCCACCGCGCCTGCATCGG | BdDMC1 | Rv |
| CROPGEN644 | TGTggtctcaacctTTCCTTCGGTTCCTCCATGC | BdBBM1 | Fw |
| CROPGEN645 | TGTggtctcatgttGGCCGTAGTCAGAAACTCAGA | BdBBM1 | Rv |
| UBI_Fw | TTTTGGTCTCAACCTGTGCAGCGTGACCCGGTCGTGC | A-ZmUBI-B cloning | Fw |
| UBI_Rv | TTTTGGTCTCATGTTTGCAGAAGTAACACCAAACAAC | A-ZmUBI-B cloning | Rv |
| CROPGEN214 | GAGATCGTGTGGGACAAGGG | ZmCas9 | Fw |
| CROPGEN215 | TGTACGCGGAGAGGACCTTA | ZmCas9 | Rv |
| **dPCR** |  |  |  |
| CROPGEN969 | CCAGTTCCCGTGCTTGAAG | BAR | Fw |
| CROPGEN970 | GGCACAGGGCTTCAAGAG | BAR | Rv |
| CROPGEN971 | TGCATGCGCACGCTCGGGTCG | BAR | Probe |
| CROPGEN889 | ATGTAGGAGGGCGTGGATAT | HPT | Fw |
| CROPGEN890 | AATGTCAAGCACTTCCGGAA | HPT | Rv |
| CROPGEN901 | TTGCATCGGCCGCGCTCCCG | HPT | Probe |
| CROPGEN972 | AAGTATGGGCATCATTCGCA | GmR | Fw |
| CROPGEN973 | ATGTTGGGAGTAGGTGGCTA | GmR | Rv |
| CROPGEN974 | GGCTCGGCCCTGACCAAGTCAAATCCA | GmR | Probe |
| CROPGEN975 | GCAAGTCAGAGGAGACGATC | zCas9 | Fw |
| CROPGEN976 | TGGCAGGTTCTTGTCGAAAT | zCas9 | Rv |
| CROPGEN977 | AGGTGGTCGACAAGGGCGCTAGCG | zCas9 | Probe |
| CROPGEN978 | ATTGCATACAACACGCTCCT | MoCRE | Fw |
| CROPGEN979 | GGTCCTGCCAATGTGGATAA | MoCRE | Rv |
| CROPGEN980 | CGCGTGAAGGACATTAGCCGCACCG | MoCRE | Probe |
